# Diffusion weighted imaging for improving the diagnostic performance of screening breast MRI: impact of apparent diffusion coefficient quantitation methods and cutoffs

**DOI:** 10.3389/fonc.2024.1437506

**Published:** 2024-12-20

**Authors:** Debosmita Biswas, Daniel S. Hippe, Andrea M. Winter, Isabella Li, Habib Rahbar, Savannah C. Partridge

**Affiliations:** ^1^ Department of Radiology, School of Medicine, University of Washington, Seattle, WA, United States; ^2^ Department of Bioengineering, College of Engineering, University of Washington, Seattle, WA, United States; ^3^ Clinical Research Division, Fred Hutchinson Cancer Research Center, Seattle, WA, United States

**Keywords:** diffusion weighted imaging (DWI), apparent diffusion coefficient (ADC), breast magnetic resonance imaging (MRI), diagnostic performance, false positives, ADC cutoff, region-of-interest (ROI)

## Abstract

**Introduction:**

Diffusion weighted MRI (DWI) has emerged as a promising adjunct to reduce unnecessary biopsies prompted by breast MRI through use of apparent diffusion coefficient (ADC) measures. The purpose of this study was to investigate the effects of different lesion ADC measurement approaches and ADC cutoffs on the diagnostic performance of breast DWI in a high-risk MRI screening cohort to identify the optimal approach for clinical incorporation.

**Methods:**

Consecutive screening breast MRI examinations (August 2014–Dec 2018) that prompted a biopsy for a suspicious breast lesion (BI-RADS 4 or 5) were retrospectively evaluated. On DWI, ADC (b=0/100/600/800s/mm^2^) measures were calculated with three different techniques for defining lesion region-of-interest (ROI; single slice(‘2D’), whole volume(‘3D’) and lowest ADC region(‘hotspot’)). An optimal data-derived ADC cutoff for each technique was retrospectively identified to reduce benign biopsies while avoiding any false negatives, inherently producing cutoffs with 100% sensitivity in this particular cohort. Further, diagnostic performance of these measures was validated using two prespecified ADC cutoffs: 1.53x10^-3^mm^2^/s from the ECOG-ACRIN A6702 trial and 1.30x10^-3^mm^2^/s from the international EUSOBI group. Diagnostic performance was compared between ADC maps generated with 2(0/800s/mm^2^) and 4(0/100/600/800s/mm^2^) b-values. Benign biopsy reduction rate was calculated (number of benign lesions with ADC >cutoff)/(total number of benign lesions).

**Results:**

137 suspicious lesions (in 121 women, median age 44 years [range, 20-75yrs]) were detected on contrast-enhanced screening breast MRI and recommended for biopsy. Of those, 30(21.9%) were malignant and 107(78.1%) were benign. Hotspot ADC measures were significantly lower (p<0.001) than ADCs from both 2D and 3D ROI techniques. Applying the optimal data-derived ADC cutoffs resulted in comparable reduction in benign biopsies across ROI techniques (range:16.8% -17.8%). Applying the prespecified A6702 and EUSOBI cutoffs resulted in benign biopsy reduction rates of 11.2-19.6%(with 90.0-100% sensitivity) and 36.4-51.4%(with 70.0-83.3% sensitivity), respectively, across ROI techniques. ADC measures and benign biopsy reduction rates were similar when calculated with only 2 b-values (0,800 s/mm^2^) versus all 4 b-values.

**Discussion:**

Our findings demonstrate that with appropriate ADC thresholds, comparable reduction in benign biopsies can be achieved using lesion ADC measurements computed from a variety of approaches. Choice of ADC cutoff depends on ROI approach and preferred performance tradeoffs (biopsy reduction vs sensitivity).

## Introduction

Breast cancer is the most common type of cancer and second leading cause of cancer deaths in women in the United States (1). Timely detection of cancer can lead to better treatment outcomes and higher survival rates for patients, making breast cancer screening a crucial aspect of women’s health. It is well-established that breast MRI offers superior sensitivity for detecting breast cancer versus other clinical breast imaging techniques and is therefore recommended for screening of high risk women (2–4). The high sensitivity of breast MRI relies on injection of intravenous contrast to identify areas of suspicious vasculature, commonly associated with breast malignancies. Dynamic contrast enhanced breast MRI (DCE) provides high sensitivity (> 85%) for breast cancer detection but suffers from moderate specificity, resulting in unnecessary biopsies that cause needless expense, inconvenience, discomfort and emotional distress for the patient (5, 6). Diffusion weighted imaging (DWI) is a non-contrast functional MRI technique that provides information based on microscopic movement of water molecules in tissues and allows an indirect assessment of tissue microstructure and cellularity. Breast malignancies tend to restrict diffusion and DWI has shown clear potential to increase breast MRI diagnostic specificity when used along with DCE.

The apparent diffusion coefficient (ADC), derived from DWI, is commonly used to quantify *in vivo* diffusion. Numerous studies have reported the utility of the metric for distinguishing between benign and malignant breast findings, suggesting ADC cutoff values could be safely used to downgrade suspicious enhancing lesions and avoid unnecessary biopsies (7–12). However, diffusion-weighted MRI is not yet incorporated into the Breast Imaging Reporting and Data System (BI-RADS) (13), and more data are needed to refine optimal methods for clinical implementation, particularly regarding quantitation of lesion ADC. Approaches to measure lesion ADC values vary and emphasize different aspects of the tumor microstructure. Choice of region-of-interest (ROI) sampling methods capture different aspects of the lesion (e.g., whole volume of the lesion to comprehensively measure the entire tumor versus ‘hotspot’ for peak cellularity) (9, 14). ADC is most commonly reported as the mean value across the lesion, measured using a manually defined ROI from a single slice. Alternative approaches of obtaining lesion ADC values include utilizing multiple (more than 2) b-values to compute the ADC map, using a nonzero minimum b-value to reduce confounding perfusion effects in ADC calculation, segmenting the whole 3D volume of the tumor across multiple slices to better account for cellular and microstructural heterogeneity across the abnormality (15), and measuring just the subregion of greatest diffusion restriction within the tumor potentially reflecting highest cellularity and proliferation within the tumor (16).

Despite evidence of ADC as a valuable biomarker for diagnosing breast cancer from multicenter prospective (10) and retrospective studies (17), implementation of DWI into routine clinical interpretations is still a work in progress. Lack of standardization of acquisition protocols and variability in ROI definition techniques and study population have resulted in a broad range of reported ADC values and diagnostic thresholds, hindering clinical integration of DWI as a screening tool. Most of the prior studies evaluated data derived from patients who received breast MRI for diagnostic purposes (to evaluate symptomatic breast tumors, abnormalities detected on other imaging modalities, or to evaluate extent of disease for known cancers) rather than for asymptomatic screening. Lesions detected in screening breast MRI exams are usually smaller and may not exhibit the same characteristics as symptomatic breast tumors, which have been used to determine the ADC cutoffs in many prior studies.

Therefore, this study aimed to investigate how different methods of measuring lesion ADC values affect the diagnostic performance of breast MRI in a high-risk screening cohort. To our knowledge, no prior research has focused exclusively on lesions identified through screening MRI. This distinctive cohort allows us to identify the most effective measurement approach for clinical incorporation of DWI in breast screening.

## Materials and methods

### Participants

The institutional review board approved this single academic medical center retrospective study (Fred Hutchinson Cancer Center institutional review no. 7339). Requirement for informed consent was waived for reviewing clinical images and medical records. Consecutive screening breast MRIs between May 2015 and December 2018 with a biopsy recommendation (BI-RADS 4 or 5 assessment) followed by definitive biopsy outcome were included in this study. Medical records were reviewed to determine two year follow-up for lesions with benign pathology on biopsy. All breast MRIs were prospectively interpreted by one of several fellowship-trained breast radiologists (including HR, with over 10 years of breast imaging experience). Over this timeframe, DWI was not generally used for BI-RADS assessment due to a lack of consensus on an ADC threshold to obviate biopsy. Lesion outcomes were classified as benign or malignant based on pathology reports after breast biopsy or excision. A subset of participants in our study (*n* = 108) were previously described in another study validating the diagnostic performance of point of care (recorded in the clinic) ADC measures of MRI detected breast lesions using pre-specified cutoffs (11). In this study, we evaluated the effects of different b-values and ROI segmentation techniques on ADC performance for reducing unnecessary biopsies in breast screening exams.

### MRI acquisition

All breast MRI examinations were acquired on a 3T MR scanner (Achieva Tx; Philips Healthcare, Best, Netherlands) with a dedicated 16-channel breast coil. Images were acquired in the axial orientation, and each exam included T2-weighted, DWI, and DCE-MRI sequences, in accordance with American College of Radiology (ACR) breast MRI accreditation and European Society of Breast Imaging (EUSOBI) breast DWI guidelines (18), and following the ECOG-ACRIN A6702 DWI protocol (10) (full protocol in **Table 1**). Onboard software provided by the scanner manufacturer was used for both spatially registering the DW images across b-values to correct for patient motion and eddy current effects.

**Table 1:** MRI protocol parameters.

	DWI	T2-weighted	DCE
Sequence type	Diffusion-weighted spin echo, echo planar imaging (DW SE-EPI)	Turbo spin echo (TSE)	Fast Field Echo (FFE)
2D or 3D sequence	2D	2D	3D
Slice orientation	Axial	Axial	Axial
Laterality	Bilateral	Bilateral	Bilateral
Phase direction	A/P	R/L	R/L
FOV	360 mm x 360 mm	240 mm x 360 mm	360 mm x 360 mm
In-plane Resolution	1.8 mm x 1.8 mm	1 mm x 1 mm	0.5 mm x 0.5 mm
Slice thickness	4 mm	3 mm	1.3 mm
Fat-suppression	SPAIR	SPAIR	SPAIR
TR	5000 ms	5000 ms	5.95 ms
TE	60 ms	60 ms	3 ms
Echo Train Length	67	N/A	N/A
Flip Angle	90 degrees	90 degrees	10 degrees
b-values	0, 100, 600, 800, 1000 s/mm^2^	N/A	N/A
Number of slices	30	~60; Variable; complete bilateral coverage	~150/ Variable; complete bilateral coverage
Slice Gap	No gap	No gap	No gap
Parallel imaging factor	3 Phase	3.1 Phase	2.7 Phase, 2 Slice
No. of averages	2 (b=0, 100), 4 (b=600, 800), 6 (b=1000)	1	1
Contrast injection	N/A	N/A	Intravenous injection of 0.1mmol/kg body-weight gadoteridol
Sequence acquisition time	4:30 minutes	2:45 minutes	2:54 mins per phase, 12 mins total (1 pre, 3 post-contrast phases with k0 at ~2, 5, and 8 minutes after contrast injection)
Diffusion Gradient Parameters			
Amplitude Duration Separation #Directions	22.52 mT/m 13 ms 21 ms 3	N/A	N/A

SPAIR, Spectral attenuated inversion recovery. N/A, Not Applicable.

### Image analysis

For the primary analysis, ADC maps were computed using a classic monoexponential decay model and least squares fitting of the signal decay across all b-values up to b = 800 s/mm^2^ (b =0, 100, 600, 800 s/mm^2^) as recommended by EUSOBI consensus for standardized reporting of breast ADC values (18, 19). The following equation was used


$$S_{b}=\;S_{0}\;*\;e^{-b*ADC}$$

where S_b_ is the DWI intensity signal at weighting b, S_0_ is the signal intensity with no diffusion weighting and ADC expressed in mm^2^/s. ADC maps were also computed using only 2 b-values (*b*=0, 800 s/mm^2^) for secondary analysis. Lesion ROIs were defined by a researcher (DB) guided by a fellowship trained radiologist (AW) who did not participate in the prospective reads, all blinded to biopsy outcomes. Using a semi-automated threshold-based software tool to avoid fat and fibroglandular tissue developed in MATLAB (Mathworks, Natick, MA) (20), lesion ROIs were defined for a single representative slice (‘2D’) and whole tumor volume across multiple slices (‘3D’) on the b = 800 s/mm^2^ images and then propagated to ADC maps. For lesions measurable only on a single slice, the 2D and 3D measurements will be the same. A subregion (9 -16 contiguous pixels, depending on lesion morphology) within the 3D lesion ROI producing the lowest mean ADC value was automatically selected by the software as the ‘hotspot’, following consensus recommendations (18). For each lesion ROI, the mean ADC of all voxels was calculated for primary analysis, while other histogram metrics (minimum, maximum, standard deviation, etc) were also calculated for 3D ROIs.

### ADC thresholds

For optimal clinical integration and patient safety, our study focused on ADC cutoffs that could reduce false positives while still maintaining high sensitivity (minimizing false negatives). ADC cutoffs were retrospectively determined for each of the ADC measurement techniques based on the highest ADC observed among malignant lesions, as previously described (10). For each ADC technique, lesions with ADC measures above the cutoff would be considered probably benign and avoid biopsy. These optimal data-derived ADC cutoffs will inherently achieve 100% sensitivity in the current dataset because they were selected retrospectively, though 100% sensitivity may not be achieved with the same cutoffs in another cohort. The primary purpose of selecting these ADC cutoffs was to enable comparison of biopsy reduction rates of the three ROI techniques at a comparable and clinically relevant operating point, where sensitivity is held fixed at 100%. We further evaluated the performance of two previously proposed ADC cutoffs: 1) 1.53 x 10^-3^ mm^2^/s determined by the ECOG ACRIN A6702 multicenter study (10) and 2) 1.30 x 10^-3^ mm^2^/s recommended by EUSOBI consensus guidelines (18, 21) and implemented in a large prospective DWI screening trial (22).

### Statistical analysis

The analysis was performed at the lesion level. Paired comparison of mean ADC values between ROI techniques or ADC maps (based on 4 b-values vs. 2 b-values) were performed using generalized estimating equations (GEE) based regression to account for non-independence of multiple lesions from the same patient (23). ADC values were also compared between benign and malignant lesions using GEE-based regression. Diagnostic performance of each ROI measure was summarized using the area under the receiver operating characteristic curve (AUC), sensitivity (proportion of malignant lesions with ADC ≤ cutoff), and benign biopsy reduction rate (proportion of benign lesions with ADC > cutoff). Confidence intervals (CIs) were computed using the non-parametric bootstrap (24) or GEE-based regression, clustered by patient, to account for non-independence of multiple lesions from the same patient. CIs for sensitivity were calculated using the Clopper-Pearson exact method (25) due to the smaller sample size of malignant lesions and only two patients had multiple malignant lesions (two lesions each). Benign biopsy reduction rates were compared between ROI techniques using the sign test and between lesion subgroups (by lesion type or lesion size) using GEE-based regression. The performance of the data-driven thresholds was further explored using 5-fold cross-validation, where different random subsamples of patients were used to rederive cutoffs and subsequently test the cutoffs in held-out subsamples not used in selecting the cutoff. The cross-validations were repeated 1,000 times and the results were averaged. Statistical significance was defined as two-sided p< 0.05. All analyses were performed using R version 4.0.3.

## Results

During the study period, 2329 screening breast MRI examinations were performed and 137 BI-RADS category 4/5 lesions were detected in 116 women (median age, 46 years, range [20-75 years]) who underwent biopsy. Pathologic assessment revealed 30 malignancies (21.9% of lesions [30/137]; 12 invasive ductal carcinoma, 13 ductal carcinoma *in situ* [DCIS], 4 invasive lobular carcinoma [ILC], and 1 malignant phyllodes tumor) and 107 benign (including 3 high-risk lesions with atypical ductal hyperplasia, lobular carcinoma *in situ*, and atypical lobular hyperplasia) lesions. None of the benign lesions upgraded to malignancy within the two year follow up period. The median size of the lesions was 8 mm (range: 3 – 76 mm), while 85% (20/137) of lesions were only measurable on a single slice due to small size or avoidance of partial volume averaging effects. Sixty-six lesions were non mass enhancements (NME) and 71 were masses (**Table 2**).

**Table 2:** Subject and lesion characteristics.

	N (%) or Median (Range)
Women (N total)	116
Lesions (N total)	137
Mean Age (years)	46 (20-75)
Race	
White	100 (86.2%)
Asian	7 (6.0%)
Black	2 (1.7%)
American Indian / Alaskan Native	1 (0.9%)
Native Hawaiian/Other Pacific Islander	1 (0.9%)
Unknown	5 (4.3%)
Ethnicity	
Hispanic/Latino	2 (1.7%)
Not Hispanic/Latino	107 (92.2%)
Unknown	7 (6.0%)
Primary MRI Screening Indication	
Personal History	35 (30.2%)
Genetic Mutation/Family History	71 (61.2%)
Other (eg, prior atypia diagnosis)	10 (8.6%)
Menopausal Status	
Pre	62 (53.4%)
Post	54 (46.6%)
Lesion size*	
≤10mm	67 (48.9%)
>10mm	70 (51.1%)
BI-RADS Assessment*	
Category 4	135 (98.5%)
Category 5	2 (1.5%)
Lesion type*	
Mass	71 (51.8%)
NME	66 (48.2%)
Method of biopsy*	
MRI guided needle biopsy	100 (73%)
Ultrasound guided biopsy	35 (26%)
Stereotactic biopsy	2 (1%)
Pathology Outcome*	
Malignant	30 (21.9%)
Invasive ductal carcinoma Ductal carcinoma in situ Invasive lobular carcinoma Malignant phyllodes	13 12 4 1
Benign	107 (78.1%)

*Calculated at lesion level (N = 137), otherwise at patient level (N = 116).

The mean and standard deviation ADC measures for each of the ROI definition techniques were 1.27 ± 0.35, 1.26 ± 0.35, and 1.16 ± 0.36 x 10^-3^ mm^2^/s for 2D, 3D, and hotspot, respectively. Pairwise comparisons revealed that hotspot ADC measures were significantly lower than 2D and 3D segmentations (mean ADC difference = 0.09 x 10^-3^ (mm^2^/s), respectively, p<0.001 for both) while there was only a small, but statistically significant, difference in ADC measures between 2D and 3D segmentations (mean ADC difference = 0.01 x10^-3^ mm^2^/s, p = 0.020).

**Figure 1:**
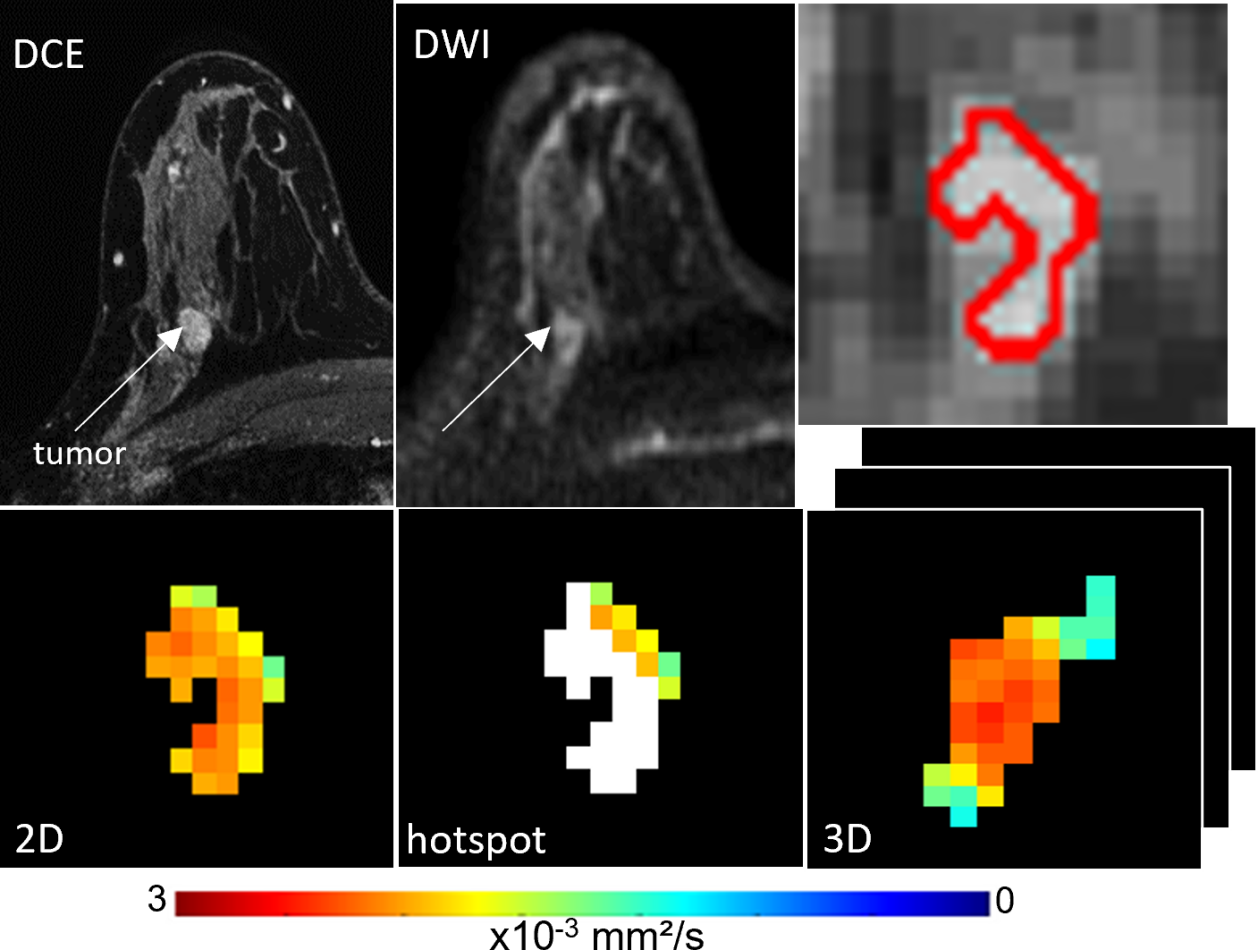
ADC measures of a BI-RADS 4 11 mm mass detected in a 41-year-old woman who underwent screening MRI. Lesion ADCs calculated using the different ROI techniques were 2.12, 1.84, and 1.97 x10^-3^ mm^2^/s for 2D, hotspot, and 3D ROIs, respectively. On biopsy, it was found that the lesion was benign breast tissue with focal fibrocystic changes.

**Figure 2:**
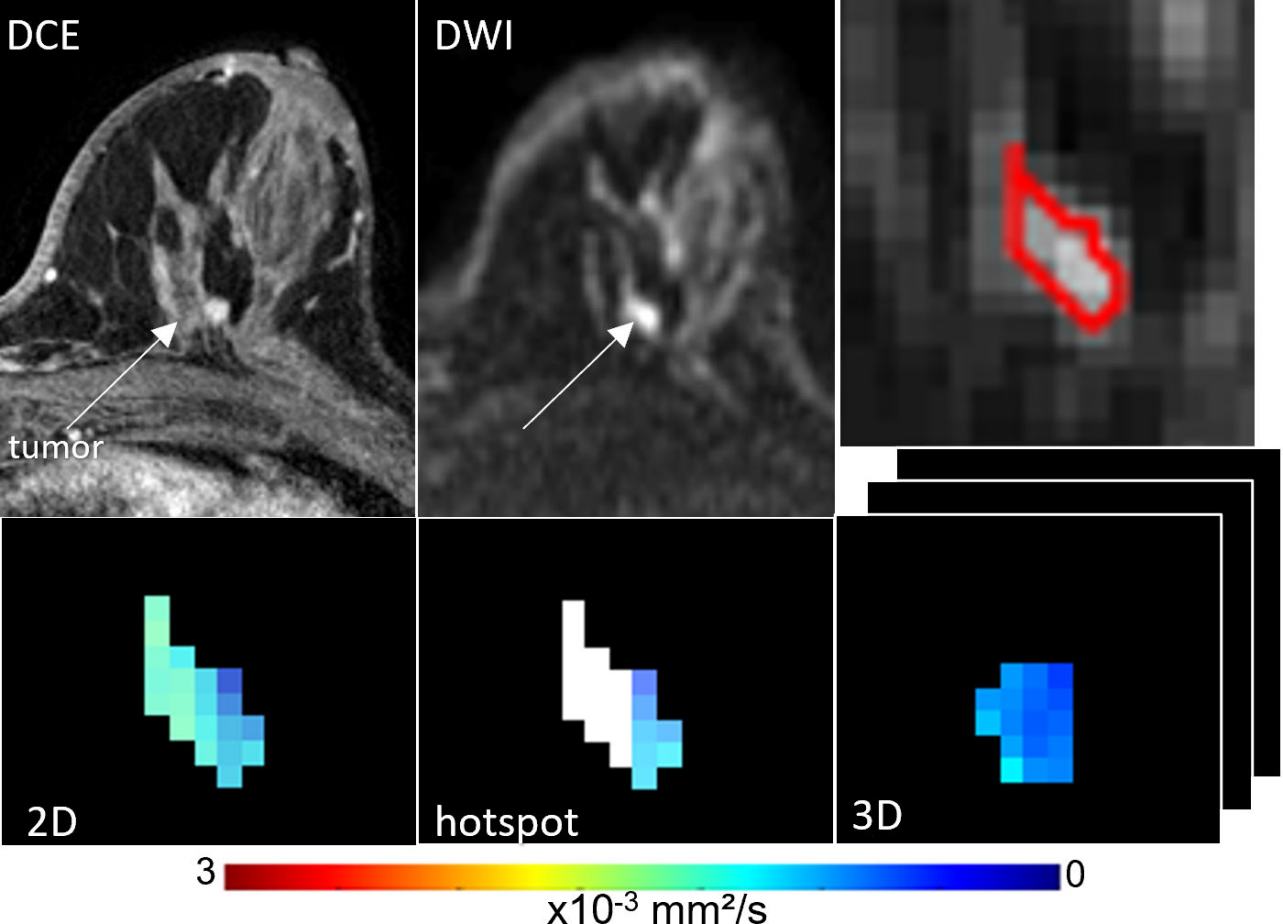
ADC measures of a BI-RADS 4 6 mm mass detected in a 55-year-old woman who underwent screening MRI. Lesion ADCs calculated using the different ROI techniques were 1.21, 1.17 and 1.22 x10^-3^ mm^2^/s for 2D, hotspot, and 3D ROIs, respectively. The lesion was invasive ductal carcinoma on biopsy.

Mean ADC measures were significantly higher for benign (range, 1.21 to 1.31 x10^-3^ mm^2^/s) versus malignant lesions (0.97 to 1.11 x10^-3^ mm^2^/s) by all three ROI techniques (p< 0.001 for each, examples, **Figures 1**, **2**). AUCs for predicting malignancy were similar for the three ROI techniques (2D: 0.66 [95% CI: 0.55-0.77], 3D: 0.67 [95% CI: 0.56-0.77], hotspot: 0.68 [95% CI: 0.57-0.79], **Table 3**). No other histogram metrics measured from the ROIs demonstrated improved performance over mean ADC (**Supplementary Table 1**).

**Table 3:** ADC for differentiating benign and malignant lesions using different region of Interest (ROI) techniques.

ROI Technique	ADC measures Mean ± SD (x10^-3^ mm^2^/s)		P-value	AUC (95% CI)
	Malignant N = 30	Benign N = 107		
2D	1.11 ± 0.29	1.31 ± 0.35	<0.001	0.66 (0.55-0.77)
3D	1.10 ± 0.29	1.31 ± 0.35	<0.001	0.67 (0.56-0.77)
Hotspot	0.97 ± 0.32	1.21 ± 0.35	<0.001	0.68 (0.57-0.79)

ADC, apparent diffusion coefficient; AUC, area under the curve; CI, confidence interval; ROI, region of interest.

### Data-derived ADC thresholds and diagnostic performance

**Figure 3:**
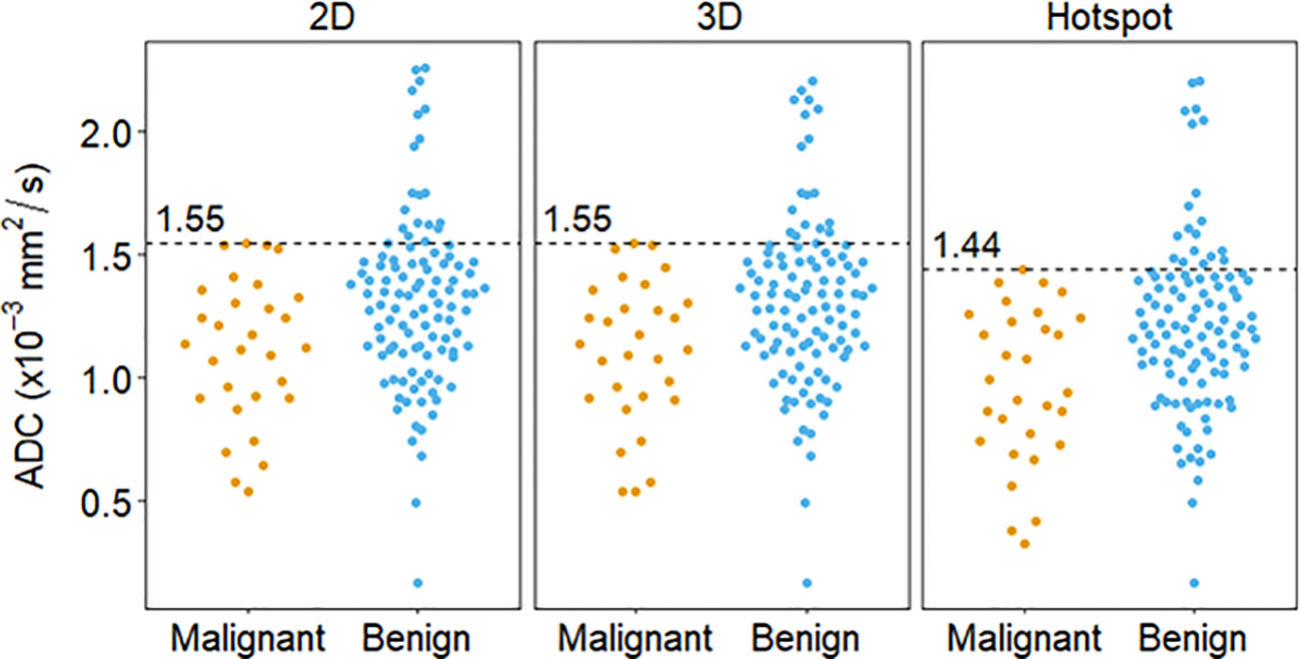
Distribution of lesion ADC measures calculated via different ROI techniques and the corresponding cutoff (dashed lines), derived using the highest malignant ADC value (100% sensitivity).

**Figure 4:**
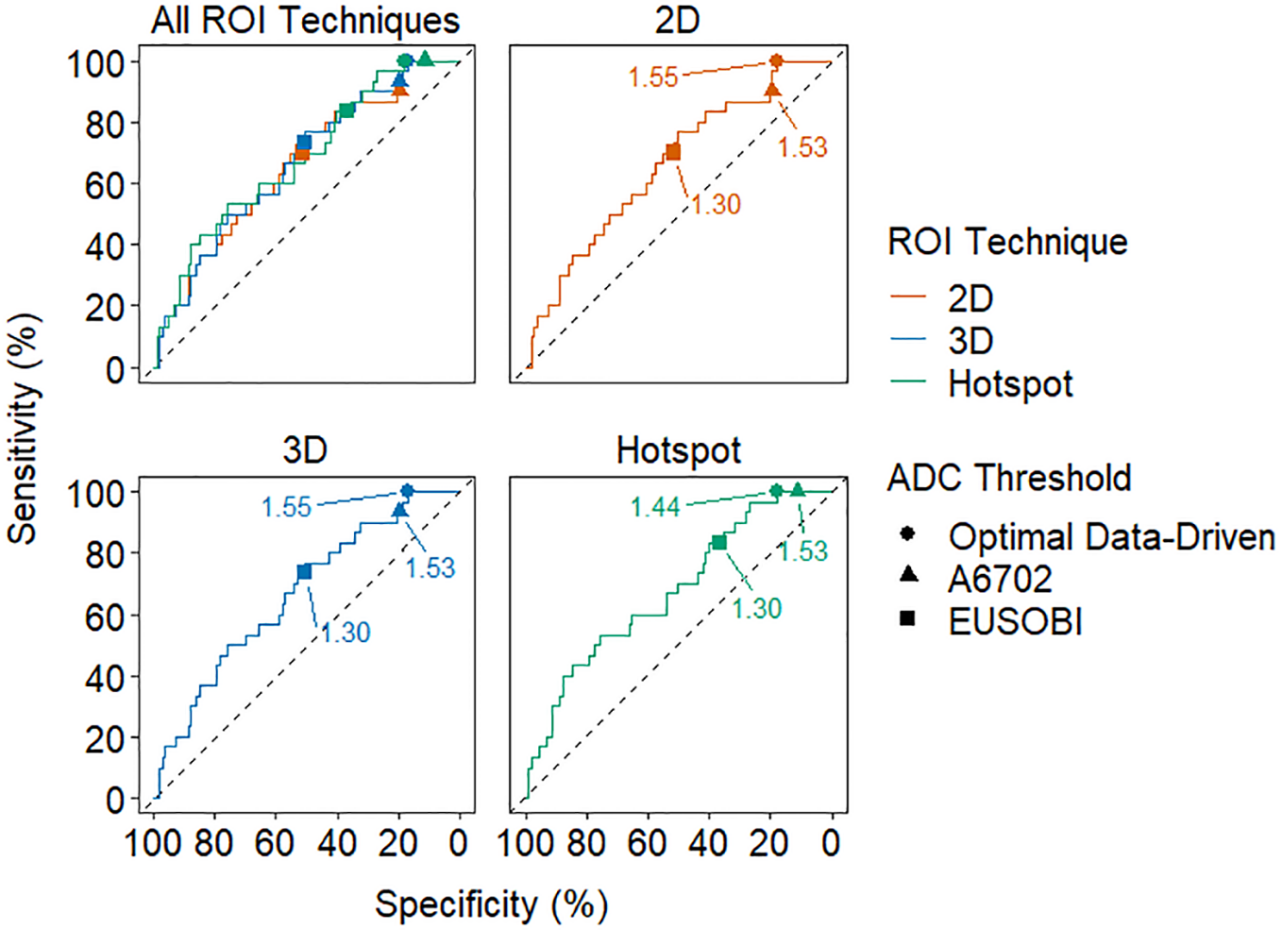
Receiver operating characteristic (ROC) curves for each ROI technique. The optimal data-driven threshold for each technique is marked with a circle on each curve and the A6702 and EUSOBI thresholds are marked with a triangle and square, respectively. The thresholds are labeled in units of 10^-3^ mm^2^/s.

The optimal ADC cutoffs derived from the data (producing 100% sensitivity) resulted in the same cutoff value for 3D and 2D ROIs (1.55 x 10^-3^ mm^2^/s) while the cutoff for hotspot was lower (1.44 x 10^-3^ mm^2^/s) (**Figures 3**, **4**; **Table 4**). Applying these data-derived ADC cutoffs resulted in a 17.8% (19/107) reduction in benign biopsies using 2D (95% CI: 10.4-25.1%) and hotspot ROIs (95% CI: 10.0-25.5%), and 16.8% (18/107, 95% CI: 9.6-24.0%) for 3D ROIs (p > 0.99 for each pairwise comparison between ROI techniques).

**Table 4:** Optimal data-derived ADC cutoffs maintaining 100% sensitivity.

ROI Technique	Optimal ADC Cutoff	Benign Biopsy Reduction Rate		
	(x10^-3^ mm^2^/s)	No.	Estimate	(95% CI)
2D	1.55	19/107	17.8%	(10.4, 25.1%)
3D	1.55	18/107	16.8%	(9.6, 24.0%)
Hotspot	1.44	19/109	17.8%	(10.0, 25.5%)

ADC, apparent diffusion coefficient; CI, confidence interval; ROI, region of interest.

Performance of the data-derived cutoffs based on repeated cross-validations was explored in **Supplementary Table 2**. Average sensitivity estimated from held-out subsamples not used to derive cutoffs ranged from 95.9% to 96.0% across ROI techniques. Average benign biopsy reduction rates across the held-out subsamples were similar to their values based on all of the data, ranging from 17.3% to 19.6% across techniques.

### Stratified performance of ADC measure

Stratifying by size, no significant differences were observed in benign biopsy reduction rates between larger lesions (19.6% [10/51] to 21.5% [11/51]) and smaller lesions (14.3% [8/56] to 16.1% [9/56]) for each ROI technique (p > 0.36 for each) (**Table 5**). Similarly, there were no significant differences in performance between ROI techniques for either larger lesions (p > 0.99 for each pairwise comparison) or smaller lesions (p > 0.99).

**Table 5:** Subgroup analysis of ADC performance by lesion type and size.

		Benign Biopsy Reduction Rate		
Subgroup	ROI Technique	No.	Estimate	(95% CI)
Masses	2D	11/60	18.3%	(7.8, 28.9%)
	3D	10/60	16.7%	(6.4, 26.9%)
	Hotspot	9/60	15.0%	(5.3, 24.7%)
NMEs	2D	8/47	17.0%	(6.5, 27.6%)
	3D	8/47	17.0%	(6.5, 27.6%)
	Hotspot	10/47	21.3%	(8.9, 33.6%)
Lesion size ≤ 10 mm	2D	8/56	14.3%	(4.2, 24.4%)
	3D	8/56	14.3%	(4.2, 24.4%)
	Hotspot	9/56	16.1%	(5.6, 26.6%)
Lesion size > 10 mm	2D	11/51	21.6%	(10.4, 32.8%)
	3D	10/51	19.6%	(8.8, 30.4%)
	Hotspot	10/51	19.6%	(9.4, 29.8%)

ADC, apparent diffusion coefficient; CI, confidence interval; NME, non-mass enhancement; ROI, region of interest.

Stratifying by lesion type, benign biopsy reduction rates from each ROI technique were comparable between NMEs (17.0% [8/47] to 21.2% [10/47]) and masses (15.0% [9/60] to 18.3% [11/60], p > 0.44 for each) (**Table 5**). There were no significant differences in performance using the three ROI approaches by lesion type. For masses: 2D ROI (18.3% [11/60]), hotspot (15.0% [9/60]) and 3D ROI (16.7% [10/60]), (p > 0.62 for each pairwise comparison) and for NMEs: 2D and 3D ROI (17.0% [8/47], hotspot (21.2% [10/47], (p = 0.69).

### Diagnostic performance of prespecified cutoffs

*A6702 cutoff (1.53 x 10^-3^ mm^2^/s):* This cutoff was determined in the A6702 trial to reduce biopsies while prioritizing sensitivity, with lesion ADC values generated using 4 b-values (same b-values as this study) and a 3D ROI approach. In this dataset, applying the A6702 cutoff to ADC values measured using the same approach as A6702 resulted in 19.6% reduction (21/107, 95% CI: 12.1-27.1%) in benign biopsies with 93.3% sensitivity (28/30, 95% CI: 77.9-99.2%). Results were similar for 2D ROI measures, with 19.6% reduction in benign biopsies (21/107, 95% CI: 12.1-27.1%) and 90.0% sensitivity (27/30, 95% CI: 73.5-97.9%). For hotspot ROIs, the A6702 cutoff was notably higher than data derived optimal ADC 100% sensitivity cutoff (1.44 x 10^-3^ mm^2^/s) and avoided fewer benign biopsies (11.2% [12/107] vs. 17.8% [17/107]).

*EUSOBI cutoff (1.3 x 10^-3^ mm^2^/s):* The EUSOBI working group recommends using a focused ROI in the area of lowest ADC within the enhancing lesion, similar to the hotspot ROI approach in this study. Applying the EUSOBI cutoff to hotspot ROI ADC measures in this dataset led to a 36.4% reduction in benign biopsies (39/107, 95% CI: 27.2-45.7%) and 83.3% sensitivity (25/30, 95% CI: 65.3-94.4%). However, applying the EUSOBI cutoff to 2D and 3D ROI measures resulted in a very high reduction in benign biopsies (50.5% [54/107] and 51.4% [55/107], respectively) but substantially lowered sensitivity (70.0% [21/30] and 73.3% [22/30]).

### Secondary analysis of ADC mapping using two vs four b-values

Pairwise comparisons revealed that ADC measures computed from only 2 b-values (0, 800 s/mm^2^) were not significantly different from measures computed using all four b-values (0, 100, 600, 800 s/mm^2^) on average (|mean ADC difference|< 0.01 x10^-3^ mm^2^/s for each pairwise comparison between ROI techniques, p > 0.17 for each). Similarly, diagnostic performance of ADC measures computed from 2 b-values were almost identical to that from ADC measured computed from 4 b-values for all ROI techniques (**Table 6**). For example, the benign biopsy reduction rates from the A6702 cutoff were 19.6% vs. 19.6% (4 b-values vs. 2 b-values) for 2D ROIs and 19.6 vs. 18.7% for 3D ROIs and benign biopsy reduction rates from the EUSOBI recommended cutoff were 36.4% vs 34.6% for hotspot ROIs.

**Table 6:** Performance of different ADC measures using prespecified cutoffs.

		A6702 ADC Threshold (1.53 x 10^-3^ mm^2^/s)						EUSOBI ADC Threshold (1.3 x 10^-3^ mm^2^/s)					
		Sensitivity			Benign Biopsy Reduction Rate			Sensitivity			Benign Biopsy Reduction Rate		
ADC Map	ROI Technique	No.	Est.	(95% CI)	No.	Est.	(95% CI)	No.	Est.	(95% CI)	No.	Est.	(95% CI)
4 b-values	2D	27/30	90.0%	(73.5, 97.9%)	21/107	19.6%	(12.1, 27.1%)	21/30	70.0%	(50.6, 85.3%)	55/107	51.4%	(41.7, 61.1%)
	3D	28/30	93.3%	(77.9, 99.2%)	21/107	19.6%	(12.1, 27.1%)	22/30	73.3%	(54.1, 87.7%)	54/107	50.5%	(40.7, 60.2%)
	Hotspot	30/30	100.0%	(88.4, 100.0%)	12/107	11.2%	(4.8, 17.6%)	25/30	83.3%	(65.3, 94.4%)	39/107	36.4%	(27.2, 45.7%)
2 b-values	2D	30/30	100.0%	(88.4, 100.0%)	21/107	19.6%	(12.1, 27.1%)	21/30	70.0%	(50.6, 85.3%)	55/107	51.4%	(41.9, 60.9%)
	3D	29/30	96.7%	(82.8, 99.9%)	20/107	18.7%	(11.3, 26.1%)	21/30	70.0%	(50.6, 85.3%)	55/107	51.4%	(41.7, 61.1%)
	Hotspot	30/30	100.0%	(88.4, 100.0%)	12/107	11.2%	(4.8, 17.6%)	25/30	83.3%	(65.3, 94.4%)	37/107	34.6%	(25.1, 44.1%)

ADC, apparent diffusion coefficient; CI, confidence interval; Est., Estimate; ROI, region of interest.

## Discussion

Suspicious enhancement of normal parenchymal tissue and benign tumors on DCE MRI leads to benign findings in as many as four in five screening-MRI prompted biopsies. Reducing false positives and unnecessary biopsies is of high importance due to the growing utilization of breast MRI for screening women with elevated breast cancer risk. At the same time, maintaining the high sensitivity of breast MRI is critical to ensure its value for early detection of disease. Although many studies have shown a clear potential of utilizing DWI for improving the diagnostic performance of breast MRI with minimum increase in cost and scan time, it has not yet been incorporated into BI-RADS. One challenge lies in determining the optimal approach for standardized integration of DWI in the clinic. Therefore, our study investigated the effect of various ADC measurement approaches and ADC cutoffs (data derived and prespecified) on performance to reduce unnecessary breast biopsies in lesions detected on breast MRI screening exams. Overall, our results using data-derived cutoffs showed that a variety of ADC measurement techniques could significantly distinguish benign and malignant breast lesions (AUCs 0.66 – 0.68) and reduce the rate of unnecessary biopsies (by 17% to 18%) of conventional breast MRI without missing any cancers. Results using prespecified cutoffs further illustrated the importance of performing lesion ADC measurements consistent to that by which the cutoffs were derived in order achieve maximal performance.

Selection of ADC threshold depends on clinical preferences regarding tradeoffs between sensitivity and specificity. Many prior studies have defined an ADC cutoff by equally optimizing sensitivity and specificity (26–28). While a false positive finding can lead to further testing (biopsy) and unnecessary emotional distress to the patient, a false negative is potentially more detrimental to patient safety as it could delay diagnosis, allowing the cancer to progress. Therefore, our study selected thresholds to maximize sensitivity, at the cost of reduced specificity, by using the highest malignant lesion ADC as the cutoff (resulting in no false negatives; 100% sensitivity) for safer adoption into clinical workflows. However, it is important to acknowledge false negatives are virtually unavoidable when applying data derived thresholds on ‘new’ datasets, as illustrated by cross-validation testing in our study. Use of a more conservative inflated ADC cutoff to keep sensitivity high may be warranted in clinical practice [e.g., a 10% inflated cutoff was proposed in the ECOG-ACRIN 6602 trial (10)]. Regarding ROI approach, we found similar biopsy reduction rates could be achieved using 2D, 3D or hotspot lesion ADC values in MRI detected lesions. However, hotspot ADC measures were systematically lower and required a lower cutoff value vs. 2D to achieve equal performance. While the choice of ROI approach did not appear to affect the performance based on lesion size (big or small), our data suggested that measuring the hotspot (lowest ADC region) of the lesion may incrementally improve diagnostic performance over 2D and 3D ROI approaches for NMEs, warranting further investigation in a larger cohort. While prior studies have found limited diagnostic value of DWI in NME lesions (29, 30), a focused ROI approach may improve diagnostic accuracy by emphasizing the tumor regions of highest cellularity (16, 31).

In addition to deriving optimal ADC cutoffs from this dataset, we also applied two pre-defined cutoffs to validate their performance for the various ADC measurement approaches. We evaluated the cutoff identified by ECOG-ACRIN A6702 multicenter trial (1.53 x 10^-3^ mm^2^/s) that prioritizes high sensitivity, which we confirmed maintained very high sensitivity (90-100%) in our dataset. This cutoff worked best for the 2D and 3D ROI approaches, with 19.6% reduction in benign biopsies (similar to the data-derived ADC cutoff), but had relatively lower diagnostic performance when using hotspot ROI measures (achieving only 11.2% reduction in benign biopsies). On the other hand, the lower EUSOBI recommended cutoff (1.3 x 10^-3^ mm^2^/s), which is being utilized in some active multicenter trials [e.g., DWIST (22)], achieved the best performance (36.4% reduction in benign biopsies and 83.3% sensitivity) using the hotspot ROI approach in our dataset because the EUSOBI cutoff led to much lower sensitivity when using the other ROI techniques (70.0% and 73.3% for 2D and 3D, respectively). Regarding the choice of b-values, fitting of DWI signal intensities using a monoexponential decay model to calculate ADC is more robust with a greater number of b-values (4 vs 2), but at the cost of longer acquisition time. Our results found no significant difference in ADC measures computed with 2 vs 4 b-values for any of the ROI approaches, with very similar diagnostic performance and reduction in biopsies. These results are consistent with previous studies that have investigated optimal b-value combinations for breast DWI (15, 18, 32) and support the use of a two-b-value combination of 0 and 800 sec/mm² for optimal efficiency.

It is well recognized that *in vivo* ADC measures are affected by the b-values used for ADC calculation (15, 33), and different scanner platforms may have varying degrees of bias due to gradient nonlinearity effects (34), while variations in other factors such as spatial resolution and field strength could introduce other effects. A strength of our study therefore was the standardized data collection, which was performed at a single institution where the MRI scanner and protocol were kept consistent over the study period. Furthermore, focused inclusion criterion of lesions detected by screening MRI only [as opposed to palpable lesions, incidental findings in cancer staging MRI exams, or problem solving exams included in prior studies (15, 17, 35)] was used to generate a unique clinically-relevant dataset to evaluate impact on MRI screening performance.

This study has several limitations. While our study focused primarily on mean ADC values for each lesion, and we did not find any advantages of using other histogram metrics, more comprehensive radiomics based measures may further improve lesion characterization. Our study followed consensus guidelines for breast tumor ADC calculation (18) and did not explore alternate b value schemas (such as using maximum b > 800 s/mm^2^ or non-zero minimum b), which would likely result in different ADC values and optimal cutoffs. Noise was not considered when calculating ADC, which may have introduced bias. Additionally, taking into account that the MRI signal follows a Rician distribution, especially in low signal-to-noise scenarios, could help make ADC estimates more consistent across different protocols, scanner hardware, and centers (36). Also, only monoexponential modeling was used for ADC map generation, while more advanced non-Gaussian, multi-compartment and other DWI modeling techniques may better characterize tissue microstructure and improve performance (37, 38). However, utilizing such advanced DWI models in breast screening applications is challenging due to limitations on scan time, small lesion sizes, and variable image quality of breast DWI in general (as ADC can be more robust to noise effects compared to other modeling parameters) (39, 40). Furthermore, all the measurements were performed offline using custom built software tools. Testing these ADC measurement techniques on clinical workstations may be needed to facilitate safe and real world implementation of DWI. Implementation of novel correction techniques during acquisition such as for gradient nonlinearity effects (34) and EPI distortions (41) are areas of future investigation to improve accuracy of ADC measures. Lastly, a larger sample size may be needed to identify subtle differences in diagnostic performance, particularly between the 2D and 3D techniques since most lesions were not measurable on multiple slices.

In conclusion, our findings demonstrate that unnecessary biopsies can be avoided for screening breast MRI exams while maintaining high sensitivity using a variety of ROI methods and b-value combinations for lesion ADC measurement. 2D, 3D and hotspot ROI approaches achieved similar rates of benign biopsy reduction using data derived ADC thresholds, which require further validation. The prespecified ECOG-ACRIN A6702 ADC cutoff worked best for 2D and 3D ROIs, whereas the lower EUSOBI cutoff was better suited for hotspot measures. Choice of ADC cutoff depends on ROI approach and preferred performance tradeoffs (biopsy reduction vs sensitivity). Shorter acquisitions with two b-values (0, 800 s/mm^2^) might be sufficient, as the diagnostic performance was similar to that of the longer four b-value (0, 100, 600, 800 s/mm^2^) acquisition. For safe and successful clinical integration of DWI to reduce biopsies, any of these ROI approaches and/or cutoffs could be applied but they need to be held consistent to achieve optimal diagnostic performance.

## Data Availability

The raw data supporting the conclusions of this article will be made available by the authors, without undue reservation.
