# Supplementary material for: Diffusion weighted imaging for improving the diagnostic performance of screening breast MRI: impact of apparent diffusion coefficient quantitation methods and cutoffs
Source: Front Oncol. 2024 Dec 20;14:1437506. doi: 10.3389/fonc.2024.1437506 (PMC11695236; doi:10.3389/fonc.2024.1437506)
Supplement: Supplementary file 1 [file DataSheet1.pdf]

Supplemental Data

A variety of histogram metrics were calculated using 3D ROIs and the results are tabulated below (Supplemental Table A1). Only the 10th percentile of ADC (17.8% [19/107] vs. 16.8% [18/107], p > 0.99) and ADC range (21.5% [23/107] vs. 16.8% [18/107], p = 0.49) had numerically higher benign biopsy reduction rates compared to mean ADC, but neither difference was statistically significant. Several other ADC heterogeneity metrics had a significantly lower biopsy reduction rate than mean ADC, including the 75th percentile (11.2% [12/107], p = 0.031), 90th percentile (9.3% [10/107], p = 0.008), max ADC (9.3% [10/107], p = 0.021), IQR (2.8% [3/107], p = 0.001), skewness (2.8% [3/106], p < 0.001), and kurtosis (4.7% [5/106], p = 0.007).

Supplemental Table A1. Performance of ADC heterogeneity metrics using the 3D ROI.

	**ADC Measures Mean ± SD ($x10^{-3}$ mm^2/s)**				**Optimal**	**Benign Biopsy**			
**ADC Metric**	**Malignant N = 30**	**Benign N = 107**	**P-value**	**AUC (95% CI)**	**Cutoff* ($x10^{-3}$ mm^2/s)**	**Reduction Rate No.**	**Est.**	**(95% CI)**	**P-value (vs. ADC Mean)**
Mean	1.10 ± 0.29	1.31 ± 0.35	<0.001	0.67 (0.56, 0.77)	1.55	18/107	16.8%	(9.6, 24.0%)	-
Minimum	0.73 ± 0.35	0.98 ± 0.39	<0.001	0.68 (0.57, 0.79)	1.28	18/107	16.8%	(9.6, 24.1%)	>0.99
10th percentile	0.82 ± 0.33	1.07 ± 0.35	<0.001	0.70 (0.59, 0.80)	1.38	19/107	17.8%	(10.4, 25.2%)	>0.99
25th percentile	0.93 ± 0.33	1.19 ± 0.35	<0.001	0.69 (0.59, 0.79)	1.46	17/107	15.9%	(8.7, 23.1%)	>0.99
Median	1.10 ± 0.30	1.31 ± 0.36	<0.001	0.67 (0.55, 0.77)	1.57	16/107	15.0%	(8.0, 21.9%)	0.50
75th percentile	1.28 ± 0.29	1.44 ± 0.37	0.006	0.63 (0.53, 0.74)	1.86	12/107	11.2%	(5.5, 17.0%)	**0.031**
90th percentile	1.40 ± 0.29	1.55 ± 0.37	0.012	0.63 (0.53, 0.74)	2.08	10/107	9.3%	(4.0, 14.7%)	**0.008**
Maximum	1.51 ± 0.30	1.65 ± 0.40	0.025	0.62 (0.52, 0.73)	2.14	10/107	9.3%	(4.1, 14.6%)	**0.021**
IQR	0.34 ± 0.19	0.25 ± 0.15	0.021	0.65 (0.54, 0.77)	0.08	3/107	2.8%	(-0.3, 5.9%)	**0.001**
Range	0.78 ± 0.27	0.67 ± 0.34	0.068	0.61 (0.50, 0.72)	0.39	23/107	21.5%	(13.8, 29.2%)	0.49
SD	0.23 ± 0.09	0.19 ± 0.10	0.023	0.65 (0.54, 0.75)	0.10	16/107	15.0%	(8.4, 21.6%)	0.85
Skewness	0.11 ± 0.75	0.04 ± 0.75	0.67	0.51 (0.39, 0.62)	-1.51	3/106	2.8%	(-0.3, 6.0%)	**<0.001**
Kurtosis	2.73 ± 1.29	2.93 ± 1.32	0.47	0.58 (0.45, 0.70)	5.78	5/106	4.7%	(0.7, 8.7%)	**0.007**

ADC = apparent diffusion coefficient; AUC = area under the curve; Est. = estimate; CI = confidence interval; ROI = region of interest; IQR = inter-quartile range; SD = standard deviation;

*Data-driven cutoffs selected to achieve 100% sensitivity. Malignancy is associated with values ≥ the cutoff for the range, SD, and skewness metrics and for values ≤ the cutoff for the other metrics.

Supplemental Table A2. Diagnostic performance of data-derived cutoffs based on 5-fold cross-validation with 1,000 replications*.

ROI Technique	Range of Optimal ADC Cutoff ($x10^{-3}$ mm^2/s)	Average Sensitivity		Average Benign Biopsy Reduction Rate	
		Estimate	(95% CI)	Estimate	(95% CI)
2D	1.41 - 1.55	96.0%	(81.8, 99.7%)	18.1%	(10.8, 25.5%)
3D	1.41 - 1.55	96.0%	(81.8, 99.7%)	17.3%	(10.1, 24.6%)
Hotspot	1.35 - 1.44	95.9%	(81.7, 99.7%)	19.6%	(11.7, 27.6%)

ADC = apparent diffusion coefficient; CI = confidence interval; ROI = region of interest.

*At each iteration, a random 20% of the patients were held out, the ADC cutoff was selected based on the largest ADC value among the remaining malignant lesions, and performance was estimated on the held-out 20% subsample. Performance was averaged across the 20% held-out sets and the 1,000 replications based on different random subsamples.
